# Ethics and the ethnography of medical research in Africa

**DOI:** 10.1016/j.socscimed.2008.02.023

**Published:** 2008-09

**Authors:** Sassy Molyneux, P. Wenzel Geissler

**Affiliations:** a KEMRI-Wellcome Trust Research Laboratories, Social Behavioural Research group, P.O. Box 230, District Hospital Grounds, Kilifi, Kenya; b Anthropology of African Bioscience, Department of Public Health and Policy, London School of Hygiene & Tropical Medicine, London, UK

**Keywords:** Research ethics, Africa, Medical research, Vulnerable populations, Human rights, Transnational research, Ethnography

## Abstract

The ethics of medical research have grown as an area of expertise and debate in recent years, with two broad approaches emerging in relation to transnational research: (1) the refinement of guidelines and strengthening of review, processes primarily to protect the right of individual research participants and strengthen interpersonal relations at the micro-level; and (2) considering more centrally, as crucial ethical concerns, the wider interests of whole populations, the functioning of research institutions, the processes of collaboration, and the ethics of inequitable international relations. We see the two areas of debate and action as complementary, and believe that social science conducted in and around transnational medical research environments can bring these two perspectives together in a more ‘situated ethics’ of research. To explore this idea for medical research in Africa, we organized a conference in December 2005 in Kilifi, Kenya. In this introduction we outline the two emerging approaches to medical ethics, summarise each of seven papers selected from the conference for inclusion in this special issue on ethics and ethnography, and finally highlight two areas of lively debate at the conference itself: the appropriateness and value of ethics guidelines and review boards for medical research; and the ethical review of social science research. Together, the papers and debates point to the importance of focusing on the ethics of relationships and on justice in both biomedicine and social science research, and on giving greater voice and visibility to the field staff who often play a crucial and under-supported role in ‘doing ethics’ in the field. They also point to the potential value of social science research on the range of relationships operating at different levels and time scales in medical research, including those surrounding community engagement activities, and the role and functioning of ethics review boards. We conclude by highlighting the ethical priority of capacity strengthening in medical research, social science and research ethics in Africa to ensure that local and national priorities and concerns are considered at both the micro and macro levels.

## Introduction

Over the past two decades, the ethics of medical research have emerged as a specific area of expertise and debate, in particular in relation to transnational research collaborations, i.e., research that is usually funded and shaped by institutions based in Europe and North America and implemented together with institutions in Latin America, Asia, Africa and Eastern Europe. Transnational research often involves enrolling relatively poor study participants in less wealthy countries (see e.g. Benatar & Singer, 2000).

Concern with the ethics of medical science is not new. It has been gaining momentum particularly since the mid-20th century in response to Nazi atrocities committed in the name of science in Germany, and the Tuskegee experiment where African Americans were deliberately denied effective treatment for syphilis (Brody, 1998). These crises contributed to the medical profession publishing several ethical codes, including the Nuremberg Code, the Declaration of Helsinki and the Belmont Report. In recent decades the debate has intensified and gained wide public attention as a result of rising concerns with economic inequities, with international human rights (particularly in relation to HIV research and access to drugs) and in response to mounting public debate about science.

## Approaches to strengthening international medical research ethics

The dominant response to emerging debates in medical research ethics has been to tighten the oversight of individual studies. Regulations and guidelines to support research ethics have been increasingly refined. There has also been a drive to expand and strengthen the capacity of bodies at local and national levels to negotiate and monitor studies, for example through ethics review committees and community advisory boards (Alberti, 2000; CIOMS, 2002; Emanuel, Wendler, Killen, & Grady, 2004; Forster, Emanuel, & Grady, 2001; Joffe, Cook, Cleary, Clark, & Weeks, 2001; Rothman, 2000; Weijer & Emanuel, 2000; Weijer, Goldsand, & Emanuel, 1999). These activities reflect a realisation that the abstract principles of existing codes are very hard to apply in practice: history, geography, culture, gender-relations and economic status can have important implications for the way in which ‘universal’ ethical principles and guidelines are prioritised and applied in different contexts. Strengthening local and national oversight is also based on the recognition that participation of competent independent specialist bodies in research-related decision-making, especially in low-income settings, is a priority for equitable transnational research collaborations.

Although many of these discussions and actions relate to concerns with wider global inequities in health and wealth, this larger context is often considered outside the immediate remit of research ethics. The focus instead is on the rights of individual research participants, and on strengthening interpersonal relations at the micro-level i.e., between researchers (and research institutions) and study communities, for example by improving and expanding informed consent procedures, and ascertaining their effectiveness in different socio-economic and cultural settings (see for example Agre & Rapkin, 2003; Lindegger et al., 2006; Molyneux, Peshu, & Marsh, 2004).

There is a growing call to move medical ethics beyond this micro-level of individual rights and interpersonal relations, and beyond matters of regulation and standardisation. The call is to consider more centrally, as crucial ethical concerns, the wider interests of whole populations, the functioning of research institutions, the processes of collaboration, and the ethics of inequitable international relations (Benatar, 1994, 2002; Benatar & Singer, 2000; Bhutta, 2002; Costello & Zumla, 2000; Jentsch & Pilley, 2003; Lachenal, in press; Wight, 2008). Here the emphasis is on recognising that research is conducted in a world of wide disparities of wealth and health, and as part of much longer term social and political processes; and that much research within this context involves vulnerable people but is not immediately applied for their benefit.

## Potential for over-prescription in medical research ethics

The two perspectives – the refinement of guidelines and strengthening of review processes, and the concern with justice in regard to wider inequities in health and wealth – are complementary and clearly described as such by most of their proponents. Nevertheless, there is concern that the increasing refinement of guidelines, and the introduction of more and more accountability mechanisms for medical research, may inadvertently narrow the scope of ethical reflection and depoliticise ethical debates. For example although many guidelines emphasise the need to adapt recommendations to local circumstances, there is a risk of guidelines being interpreted and drawn upon far more narrowly than was originally intended. The result can be detailed lists of ‘correct procedures’, similar to laboratory ‘standard operating procedures’ (SOPs); lists and check-boxes that potentially undermine rather than promote vigorous and critical ethical debate, and that may detract from rather than encourage the public negotiation of the scope and aims of science (i.e., the broader ethics of scientific knowledge).

The trend towards increasing attention to matters of detail in research ethics, such as consent form reading scores or standardised communicative gestures when dealing with study subjects, parallels similar trends in the conduct of scientific work itself. Randomised clinical trials, for instance, and the procedural standards of the pharmaceutical industry, which have gained importance in public health research in recent decades, rely on specific operational rules and institutional resources. Importantly, this particular form of ‘raised standards’ – i.e., strict regulation of science by laboratory procedures and Good Clinical Practice (GCP) – has not always been accompanied by greater public engagement and trust. Indeed, the public in Europe as well as Africa is often less optimistic about than concerned with science (see e.g. Leach & Fairhead, 2007). Particularly relevant to the African context, raised procedural standards and reliance on high-end scientific technologies imply high costs. There is therefore a growing concentration of internationally competitive research in relatively few collaborative research centres. These centres tend to be funded by transnational charities and Northern research institutions rather than African national governments. While such centres offer vital opportunities for scientific progress and for research capacity strengthening, they are also faced with new challenges regarding their relations with local health systems and the public. Thus, while the rules of doing science are defined more and more precisely, the wider direction and public good associated with science, arguably the core of scientific ethics, has become less clear.

## Research ethics and ethnography in ‘the field’

Our own view is that both of these areas of debate and action – the development, review and monitoring of adherence to ethical principles and guidelines for medical research, and the critique of the political economy within which they are developed and applied – are needed. The former without the latter turns research ethics into an apolitical instrument that obscures the social conditions of science and the social-economic determinants of health. The latter without the former provides for political discussions, but does not avail potentially vulnerable study populations the legal instruments (such as ethics codes) to defend their own best interests. Any apparent opposition in the two perspectives is particularly diminished when we move away from global academic debates towards the everyday practice of international medical research. We think that engagement of social scientists in medical research environments – both as participants and as participant observers, and not only in field settings but also in academic institutions, review boards, medical practice and policy environments – can contribute substantially to understanding the social, economic and political contexts of health. Such an understanding, as Benatar and Singer (2000) observe, is essential to the conduct of ethical research. Social research in these environments can help us to move closer towards a more ‘situated ethics’ of research. A situated ethics considers the relevance and application of ethical principles and guidelines for different studies and contexts, and takes into account the realities of complex individual, institutional and national imbalances in power and resources. Thus, it potentially brings together the micro- and macro-level perspectives outlined above.

We therefore propose a broad ethnographic engagement with the ethics of medical research, which applies the tools of social anthropology and other qualitative social science to the networks and social processes in which transnational medical science is produced. Such ethnographic research can take a range of forms and can focus on different levels of scale (see, e.g. Booth, 2004; Fairhead, Leach, & Small, 2005, 2006; Geissler, 2005; Petryna, 2006; Turnbull, 1989). The ethnographic approach derives insights from observation of and participation in the everyday conduct of social life – in our case of everyday scientific life – and is searching and open-ended. Our suggestion reiterates a point made earlier by Kleinman (1999, p. 89), who proposed ethnography as an important remedy against the dominance of “economics, decision analysis, and legal procedures” in policy and bioethics debates. We differ slightly from Kleinman's proposition in suggesting less emphasis on the tensions between ‘global’ and ‘local’ ethics (i.e., the cultural dimensions of local ‘moral worlds’), and focusing instead on the practical social relations between realms and across levels of scale. The approach is also premised upon close attention to the *relations* between different members of social networks (individuals, groups or institutions), and the tools, sites and technologies that they employ. This implies a shift away from observing defined groups or entities – as in studies of ‘community perceptions’ or ‘cultural ethics’ – and towards what happens, over time, between these entities. Moreover, an ethnographic approach to research ethics would aim to move across very different *levels of scale*, linking, for example, the everyday life of study communities, the historical processes of the societies and states they live in, transnational scientific institutions, and global flows of knowledge and resources. In our view, such an ethnographic perspective can be useful not only to the professional social scientist, but equally to any other member of the scientific community who wants to understand science and its ethics better.

## The Kilifi Conference

To explore the possibilities of such an inquiry we organised in December 2005 a one week international conference from which the papers presented in this collection were selected. This conference, jointly organised by the Health Policy Unit, London School of Hygiene and Tropical Medicine, and the KEMRI-Wellcome Trust Collaborative Research Programme in Kilifi, Kenya, and hosted by the latter, brought together a multi-disciplinary group of social scientists who have studied the conduct of medical research in Africa or worked in medical research sites in Africa, and specialists in ethics, medical research, human rights and policy. The overall aim of the conference was to explore the ‘trial community’; that is the broad social network that includes study participants, scientists, research staff, funders, academics, health care providers, government representatives and members of the public.

For this Special Issue on ‘ethics and ethnography’, we have chosen a selection of papers that discuss the relevance and ethical implications of social science research for medical research.^1^ The first three papers look at the primary, field-level social relationships involved in biomedical research: relationships between communities and research teams (Geissler, Kelly, Pool, & Imoukhuede, 2008; Gikonyo, Bejon, Marsh, & Molyneux, 2008; Marsh, Kamuya, Gikonyo, & Molyneus, 2008). The next three papers shift the focus away from the field, and towards relations between researchers and health policy makers and implementers at the national level (Gilson & McIntyre, 2008; Lairumbi et al., 2008; Theobald & Nhlema-Simwaka, 2008). These six papers together point to the ethics of medical research pertaining to all social relations within the network of the ‘trial community’, and not merely to the researcher–research participant interface. They reveal also the great need for more detailed, ethnographically inspired scrutiny of the more ‘detached’ levels of research; namely, research collaboration, review and regulation, and links to government bodies and policy and health system actors. Our collection is completed by a paper that reflects on issues related to the ethics of social science research itself. Nyambedha takes his own experiences as an anthropologist studying the impact of AIDS in Western Kenya as a starting point (Nyambedha, 2008). He reflects on the particular ethical challenges of the ethnographer's position, focusing on consent, feedback, and expectations. This paper links to points raised in Theobald and Nhlema-Simwaka regarding the ethical review of health related social sciences in Africa, highlighting institutional challenges and needs.

## Working in the field: Researchers and study participants

The first set of papers is based on a perception that current guidelines for carrying out internationally funded biomedical research in economically deprived settings have been informed by relatively little good quality empirical research. Ethics debates tend to be dominated by the views of scientists and advocates from high-income settings, or by professionals from low-income countries who have had little opportunity to engage in the actual conduct of studies. The perceptions and priorities of the diverse communities who are the subjects of research, of the local researchers and research assistants who are primarily responsible for implementing ‘ethically appropriate’ practices, and of the health workers, managers and policy makers who are so often expected to put research findings into practice, are therefore rarely heard.

Gikonyo et al.'s and Geissler et al.'s papers draw on qualitative data from a range of actors involved at the field-site level in malaria vaccine trials in Kenya and Gambia, respectively (Geissler et al., 2008; Gikonyo et al., 2008). The two papers include similar findings. Firstly, it is clear that all biomedical research is inevitably a social endeavour, with research ethics being influenced by shifting social relationships between the range of individuals and communities involved. Fieldworkers who are based in ‘the field’ face significant challenges in mediating between the very different priorities and concerns of well resourced research institutions and low-income communities. In the process, they do not simply neutrally observe and adhere to formal externally derived ethical rules, but instead play a vital, creative, and under-recognised role in research and ethics practice. Such interactions have both positive and negative implications for community members and researchers. Second, the social relationships between actors – and perceptions of and participation in studies – are based on and are continually tested by context specific concerns and interests. These concerns and interests can be difficult to predict, and extend well beyond the timescale and reach of single research activities. Giyonko et al. and Geissler et al. conclude that while formal ethical guidelines play an important role in regulating research practice, implicit day-to-day social relations and engagements between people are fundamental to the research process (see, for similar observations, Meinert, in preparation; Whyte Kaharuza, & Whyte, in press). Gikonyo et al. argue for greater attention to these social relations at a time of increasingly ambitious and stringent formal ethical standards in bioethics (Gikonyo et al., 2008). Geissler et al. concur with this viewpoint, but warn that efforts to establish ‘good relations’ should not be considered a panacea to the ethical and political dilemmas of transnational collaborative research (Geissler et al., 2008). They argue that in the face of the global political and economic inequality, and given the relative weakness of the conventional representative institutions in many economically deprived countries, new public accountability and institutional spaces are also needed. These spaces can help arbitrate the different interests of individuals and groups involved in transnational medical research, and contribute to a more equitable and democratic medical science.

Marsh et al. (2008) (see also Starling, Kamuya, Gikonyo, Molyneux, & Marsh, 2007) could be seen to respond to the last point raised by Geissler et al.: the need to establish some form of democratic representation of study communities in relation to research institutions (Marsh et al., 2008). They outline the process of involving a wide range of individuals and groups in the development and implementation of a community engagement strategy aimed at improving mutual understanding between community members and researchers in a large multi-disciplinary research programme on the Kenyan Coast. In so doing the authors contribute to the growing interest in community engagement in biomedical research, for which there is relatively little published experience. Overall, the strategy involves new and diverse opportunities for regular dialogue and interaction, and for partnership building between actors. These discussions and interactions appear to be having an impact not only on information-giving and associated materials, but also – and possibly more importantly – on institutional policy and practice. Examples of the latter include new induction, training and support requirements for all staff, including field workers, reconsidering approaches to and levels of benefit sharing between the research institute, the Ministry of Health and community members (participants and non-participants), and rethinking employment strategies. The authors also highlight a series of emerging issues and challenges ranging from the complexity of defining ‘the community’ or ‘communities’ involved, the nature of representation (who is represented by whom), the resources and flexibility required to be adequately responsive to concerns and issues raised, and shifting power imbalances between the research centre and other local communities. These challenges point to the need for ethnographic research around community engagement processes and ownership of research, in order to feed into ethics debates at both the micro, interpersonal level, and the wider institutional level.

## Shaping policy: Researchers and the health system

Lairumbi et al. (2008), Theobald and Nhlema-Simwaka (2008), and Gilson and McIntyre (2008) move a step away from the material encounters in the field and towards relationships and collaborations between researchers and other health actors at the regional and national level. Each of these papers is concerned with the widely advocated imperative for health research to generate local social value through producing knowledge for generalised health improvements, and contributes to the literature around the challenges and strategies for achieving this. In so doing they are also contributing to debates around what happens at the end of research, when the immediate material benefits that research work usually brings to the study communities come to an end, and research results with potential health policy and practice relevance are produced.

Drawing on exploratory in-depth interviews in Kenya with policy makers, researchers, policy implementers and representatives of organisations funding health reforms, Lairumbi et al. (2008) provide additional evidence of the significant and well recognised gaps in the research to policy to practice pathway, particularly for biomedical/clinical research and for national research institutions. They highlight the considerable power of the global health and research agenda to inhibit the development of a more varied or context specific local agenda; a problem exacerbated by the weakness of local health information systems, lack of local funds to support research and weak communication between policy makers and researchers, and between researchers/policy makers and those delivering health care. They also report that dissemination of findings relies heavily on traditional means – such as publication in international academic journals – that are not accessible to many local stakeholders. They point to the need for a more interactive model of information sharing between all levels of actors as opposed to the more frequently cited linear paradigm of research to policy to practice. The paper illustrates the centrality of power relations in every stage of the research process – from concept to practical application of the findings – and the importance of recognising these differentials in research practice. The authors also call for efforts to transform the inequitable relations surrounding research, through, for example, greater resources for and autonomy of local institutions over research agendas, and investment to support the mechanisms and structures to coordinate appropriate collaborative research processes. Beyond these recommendations, Lairumbi et al.'s paper also draws our attention to changing relationships between research, medicine and government. Conventional linear models of research to policy were developed when most research was funded by governments, and when government was the principal health care provider to its citizens. In the current age of parastatal research institutes and transnational science, and of bilateral and ‘big charity’ interventions in health care that often dwarf the health budgets of Southern nations, the direct link between science and politics and government has been weakened. This changed relation between medical science and the state emerges as an important, also ethical, concern deserving further historical and ethnographic study.

Theobald and Nhlema-Simwaka (2008) and Gilson and McIntyre (2008) contribute to these debates and to practical ideas and responses through their own experiences of working at the research/policy/practice interface in Malawi and South Africa, respectively. Gilson and McIntyre's paper serves as an important reminder of the complex ways and timeframes over which different types of research can influence policy and practice, and thereby achieve local social value. The authors highlight that while there is a tendency to judge the instrumental use of research as the acid test of policy impact (i.e., generating changes in behaviour and practice that may solve particular problems), the complexity of policy processes means that much research has a far longer term and more indirect influence over policy. In discussing the policy impact of a collaborative study carried out by the two local health policy and systems research institutions that they work for, Gilson and McIntyre highlight a series of inter-related factors influencing if and how research is ‘taken up’, including the policy issue, the political context, the credibility of the research and researchers involved, the dissemination of findings, and how networked with knowledge users and other researchers the individuals and institutions are. In so doing the authors provide a useful framework and set of practical suggestions for others to consider in strengthening and evaluating the benefits that accrue from research. Gilson and McIntyre also hint at the difficulty of achieving policy and practice impact by individuals and institutions external to national health policy and practice, and at the fundamental importance of ‘outsiders’ to the systems and country having strong collaborative partnerships with local and established individuals, institutions and structures (Gilson & McIntyre, 2008).

Theobald and Nhlema-Simwaka's paper, in synthesising the opportunities and challenges they have encountered in promoting the use of applied social research on TB and HIV in Malawi, also illustrates the complexity at the research–policy–practice interface (Theobald & Nhlema-Simwaka, 2008). As do Gilson and McIntyre, they highlight the need for being involved in the medium and long term in local institutions and structures which bring together the relevant stakeholders. This facilitates the development of participatory interactions with policy makers, practitioners and community members, and enables promotion and advocacy for applied social science and the uptake of findings with potentially important public health impacts. Specific opportunities and challenges they highlight for qualitative research in HIV/TB are, on the one hand, the clear need for qualitative methodologies to understand and respond to the multiple barriers that poor women and men face in accessing services, and on the other hand widespread concerns locally and globally about the validity and generalisability of qualitative methods and data. A related concern is the significant capacity constraints in the social sciences within the country and region. In line with Wight (2008), Theobald and Nhlema-Simwaka highlight that individualistic models of consultancy – where individuals are contracted out for short-term, well paid contracts – can serve to undermine rather than strengthen social science research capacity; and that this tendency is exacerbated by challenges around ownership of the products of consultancy and challenges in publication and dissemination of findings.

In exploring relationships around the research to policy to practice interface, all three papers illustrate the challenges posed by applying the ethical principles of local social value and of respect to persons and communities, in contexts of national and global disparities, and of the importance of developing institutional set-ups and relationships that contribute to transformation of these inequities. The differences between the South African and the Kenyan situation – for example in terms of the relative power of government institutions and of national scientific institutions – are noteworthy. Nevertheless, the similarities of the authors' observations and concerns with the determination of research agendas by outside institutions, and the insufficient integration into national health agendas, underline the need for intensified South–South exchanges and collaboration on these issues.

## Ethics *of* ethnography? The regulation of health-related social science research

Hoeyer, Dahlager, and Lynoe (2005) have pointed out that there tend to be less elaborate requirements and codification of research practices in the social sciences than in biomedicine. Instead, in the social sciences there is generally greater awareness of and attention to changing and conflicting interpersonal relationships, and greater concern with justice and the political implications of the research endeavour (see also e.g. American Athropological Association guidelines, 1998). Interest in relationships in the social sciences, and in the power imbalances inherent in these relations, is reflected in the issues and foci of the papers already presented, and illustrates the potential role for social scientists in contributing to the debates around the micro- and macro-level ethical issues surrounding biomedical research. However, the engagement of social scientists with the ethics of research practices of other academic disciplines also invites reflection about the ethical issues raised by carrying out such social science studies themselves.

The papers by Gilson and McIntyre (2008) and Theobald and Nhlema-Simwaka (2008) begin to do this for health policy and systems research and for applied social research respectively. Their papers illustrate the important implications that the physical and socio-political location of social scientists has in developing research ideas, over the course of conducting research, and on the impact research has on policy and practice. These papers also suggest that regardless of the aim and type of study, social science findings relevant for public health may need strong and often informal networks between knowledge users and other researchers, and ‘strategic framing’ (careful wording depending on targeted audience), to have a chance of impact.

Nyambedha's paper brings us back to the field, this time with reflections on the ethical dilemmas faced in social science research on AIDS and orphanhood in Western Kenya (Nyambedha, 2008). His moving description is a reminder of the context of extreme vulnerability within which many of us work, and the impact we have on social relationships despite extended ethnographic work, efforts to carefully explain the study and feed back the findings locally, and the absence of biomedical interventions. Nyambedha's daily experiences in the community suggest that many of the ethical principles and guidelines developed for biomedical research can also be relevant for social science research. Clearly, for much health-related social science research there is a need to carefully consider at the outset – and to continuously monitor and (re)consider throughout the research through community engagement – the risks and benefits of studies to communities and societies, and how these balance against recognised benefits to researchers themselves (i.e., salaries, publications, international travel, exposure and reputation). More fundamentally, his frank and open discussion of significantly raised expectations of individual and community level benefits as a result of being involved in his research, despite repeated efforts to explain his position, illustrate the profound challenge of doing any kind of basic research in a situation of gross poverty and suffering. It also makes very clear the personal ethical challenge of conducting scientific work in poor, vulnerable populations. This challenge cannot be resolved by ethics guidelines alone, but also requires personal moral reflexiveness and integrity.

These ethical challenges of social science research are worthy of deliberation; and arguably differ in important ways from those facing clinical researchers. The appropriateness of social science studies being subject to similar ethical review processes as biomedical studies, and often by committees more familiar with the former, was a source of lively and valuable debate at the conference (discussed in more detail below).

Theobald and Nhlema-Simwaka (2008) argue that there is a need to explore and document practical ways to build capacity and to support and mentor Research Ethics Committee (REC) members to review and appraise social science protocols (a similar point was discussed in depth by Wassenaar & Corbella 2005; see Wassenaar, 2006). They see this as essential in order to move towards a situation where RECs can act as a catalyst and support structure for quality and ethically sound social science research rather than as an impediment. They suggest two complementary strategies: advocating for social scientists to sit on RECs, and supportive capacity strengthening for REC members on how to assess quality in qualitative research protocols. Regarding the development of checklists and guidelines for the review of qualitative research, they emphasise that ‘there is a need to be critical and creative in application, because qualitative research comes in many different shapes and sizes, and has different theoretical roots’ (Theobald & Nhlema-Simwaka, 2008).

## Emerging issues from the papers and wider conference

Empirical studies regarding the ethics of biomedical research have to date focused on participant views and understandings of biomedical research, factors influencing these, and on the gossip, scandals and public scares that are so often reported around research (Fairhead et al., 2005, 2006; Geissler & Pool, 2006; Molyneux et al., 2004; Molyneux, Peshu, & Marsh, 2005; Molyneux, Wassenaar, Peshu, & Marsh, 2005; Singh & Mills, 2005; Tindana, Kass, & Akweongo, 2006). These issues remain a critical area of study and are often featured in the papers presented here. However in this collection we have also tried to move beyond these foci and to consider instead the dynamic relationships between the various actors involved in research activities at different levels; relationships that are both impacted on and influence the science and ethics of biomedical research. These are embedded in a wider context of socio-political global inequities. We see that there are important ethical dilemmas and challenges faced by all researchers in contexts of immense poverty and struggling, and that the generally stronger focus on the ethics of relationships and on justice in the social sciences is highly relevant also to biomedicine.

An emerging issue from the papers in this Special Issue (and from the wider conference) is that in considering key relationships in research, and issues of justice, field staff need to be given greater visibility and voice. Many play a crucial and often under-recognised and under-supported role in ‘doing ethics’ in the field, with important implications for the success and quality of the science itself. This suggests that field staff should not only be considered as the subjects of future research, but also that they need to be taken more seriously as collaborators and partners in research endeavours. This approach will require careful thinking around appropriate training activities and career paths for these vital staff.

What we cannot adequately convey in this collection of papers is some of the rich discussion and debate that took place at the meeting itself in Kilifi, and the insights of other ethnographers, historians, and ethicists. These participants' papers and some of the debates are presented elsewhere (Geissler & Molyneux, in preparation; Wight, 2008). Papers include discussion of the role social science reflection can play in understanding the nature and trajectory of science itself (including the contemporary dominance of the randomised controlled trial and of “evidence” based medicine), and the way in which history, and the memories of historical engagements with medical research, continue to shape health research in Africa today. At the conference, there was also lively debate on the relevance and appropriateness of biomedical research ethics, as expressed through ethical guidelines and review boards, particularly if transferred from biomedical to social science research.

## Debates around ethics guidelines and review boards for medical research

As noted in the introduction, ethics guidelines and independent review boards have been developed and refined in an effort to minimise the potential of human rights abuses in science, and to tackle global inequities in research-related wealth and power. One critique of these developments is that, rather than fostering genuinely ethical research, there is a risk that detailed rules and requirements may prevent ethical thinking; that they risk relinquishing the ethical impulse between the researcher and the researched. In other words, bureaucratic rules potentially replace responsible practice. Another argument is that rules and regulations may enable researchers, funders and institutions to side-step the more fundamental ethical issues of the politics of poverty and inequity, which should be at the core of the public health agenda.

A counter-argument to concerns about the overly bureaucratic application of rules is that well-functioning ethics review boards should not be blindly applying principles and guidelines but should rather be drawing on them in a scholarly, skilful way to ensure a balanced approach in considering each study. In so doing RECs should contribute to ethical awareness of all researchers, promote good quality research with short- or long-term local benefit, and avoid research that will exploit vulnerable individuals and communities. A counter-argument to concerns that the politics of poverty are side-stepped by guidelines and review boards is that ethics review committees should be part of local and national endeavours to challenge global inequities; in particular the imposition of inappropriate external research agendas. Furthermore, it is argued that if there are shortcomings regarding the institutional and individual capacity of RECs, then these limitations should be overcome rather than used as an argument for their irrelevance or inappropriateness. A concern regarding increasing the capacity of local RECs is that much, but not all, of the training in resource poor countries is short-term and donor-driven, with inadequate opportunity to consider application in diverse contexts. Rather than fostering critical local ethics scholarship, such training may therefore reinforce rather than transform current global disparities (see Ulrich, in press).

Clearly further arguments can be and were made at the conference on both sides of the debate. Ethnographic work on RECs – their linkages and relationships with other actors in trial communities, the challenges they face, and the successes and challenges around efforts to overcome these – could contribute usefully to such debates for different types of biomedical research in future. Moreover, these debates underline that the concept of research ethics or bioethics, its historical emergence and transformations, and the workings of regulatory frameworks, review boards and concrete ethics procedures, ought not to be taken for granted; rather than being self-evident, they constitute an important subject of ethnographic and historical research.

## Debates around ethical review of social science research

With regard specifically to the ethical review of social science research, debates at the conference highlighted the broad spectrum of research traditions and disciplines included under ‘social science’. Social scientists' epistemological bases, and the types of research they are involved in (basic or applied), can have a significant influence on their perceptions of the relevance or adequacy of biomedical ethical frameworks for their studies, and of the formal ethical review process increasingly applied to health-related research in Africa. A particular concern among many participants was that the aims and outcomes of some anthropological studies and historical research differ from those of medical science, and that although such work obviously requires careful and continuous ethical reflection, current guidelines and review bodies are inadequately equipped to check and monitor these in many cases. For example, while scientists conducting medical trials know what they want to prove or learn about from the outset, and by which methods they will achieve that goal, some anthropological enquiries enter the field with little more than a main, often theoretically orientated, question. They may not know the answers they are looking for, who exactly the informants are that will provide these answers, or under which conditions.

Another debate was around expected outputs; while some social scientists working in health research institutions have a mandate to contribute more or less directly to interventions that promote improved health, others – in particular academics in conventional university departments – are under no such professional obligation (although they might be personally committed to progressive aims including public health). They are under pressure instead to contribute to the advancement of their discipline in terms of theoretical debates and publication in respected journals. Moreover, many social scientists see their main role as critical analysts of social processes (including scientific and health policy processes) rather than as ‘social engineers’. They fear that a direct bond of social inquiry to societal interventions and outcomes – such as, for example, by meeting predetermined ‘social value’ criteria – could curtail the academic freedom that the humanities (like any other science) require to make a critical contribution to social progress. With regard to ethics regulations and institutional review systems, many social scientists fear that these might impose social values and orders upon them, which, in their view, should instead be the objects of their critical scrutiny.

A concern raised in response to these arguments was that these standpoints should be vigorously interrogated to ensure that they are not mere expressions of academic convenience or complacency, or attempts to avoid independent ethical scrutiny and debate altogether. Debates about if and how all social science research should be independently scrutinised, and whether all social science involving interactions with people should adhere to the same rules as medical research with human participants, remained unresolved during our conference. They require further reflection in the context of the recent expansion of research ethics in medicine and beyond, and at a time when transnational social science research is increasingly funded by health-related grants and conducted in relation to health and other policy orientated programmes.

## Support for capacity strengthening in science and ethics

While current mechanisms for ethical scrutiny of health-related research provoked controversial debates, there was widespread agreement among the participants at the conference around the fundamental importance of strengthening individual and institutional academic capacity within Africa and the profoundly ethical nature of this requirement. Only if institutional and personal collaborations in transnational research are equal and fair, the participants agreed, can ethically sound and democratic collaboration occur, and only if relations between (social and natural) scientists in medical research are balanced in terms of knowledge, interest and power, can a genuine partnership between researchers and study populations, and scientific bodies and society become reality. Clearly this research capacity strengthening is a priority not only for medical science, but also for the social sciences and for health research ethics. Together with ensuring that health research ultimately leads to health improvements, strengthening regional capacity to drive the health research agenda, and to develop locally appropriate review systems, should in the future ensure that local and national priorities and concerns at both the micro and macro levels are considered in the settings where research is conducted.

Many of the papers presented in this collection offer insights into the ethical complexities and challenges around carrying out biomedical and social science research in low-income settings. Several offer ideas around how to strengthen research ethics, including around improving capacity strengthening in the social sciences and in research ethics. Most papers are exploratory studies, reflections and opinion pieces, suggesting further research and thinking rather than concrete conclusions. However, we believe that, together with the wider conference debates and papers, they illustrate the potential of the social sciences to contribute to the evidence base and discussions around health research ethics. We hope that the collection will encourage a growing body of work and discussion, ideally driven by individuals and institutions from the region, that focuses both on the relevance and application of ethical principles and guidelines for different studies and contexts, and the complex individual, institutional and national imbalances in power and resources within which all research is conducted.

## Footnotes

1 Another paper from the conference has been published separately (Wight, 2008), and a further collection is currently being prepared for publication in an edited book (Geissler & Molyneux, in preparation).
